# The Class-A GPCR Dopamine D2 Receptor Forms Transient Dimers Stabilized by Agonists: Detection by Single-Molecule Tracking

**DOI:** 10.1007/s12013-017-0829-y

**Published:** 2017-11-07

**Authors:** Rinshi S. Kasai, Shuichi V. Ito, Ryo M. Awane, Takahiro K. Fujiwara, Akihiro Kusumi

**Affiliations:** 1 Institute for Frontier Life and Medical Sciences, Kyoto University, Kyoto, 606-8507 Japan; 2 Center for Meso-Bio Single-Molecule Imaging (CeMI), Institute for Integrated Cell-Material Sciences (WPI-iCeMS), Kyoto University, Kyoto, 606-8507 Japan; 3 Membrane Cooperativity Unit, Okinawa Institute of Science and Technology, Okinawa, 904-0495 Japan

**Keywords:** Dopamine receptor, G protein-coupled receptor (GPCR), Plasma membrane, Cultured cell, Single-molecule biophysics, Dimerization

## Abstract

Whether class-A G-protein coupled receptors (GPCRs) exist and work as monomers or dimers has drawn extensive attention. A class-A GPCR dopamine D2 receptor (D2R) is involved in many physiological and pathological processes and diseases, indicating its critical role in proper functioning of neuronal circuits. In particular, D2R homodimers might play key roles in schizophrenia development and amphetamine-induced psychosis. Here, using single-molecule imaging, we directly tracked single D2R molecules in the plasma membrane at a physiological temperature of 37 °C, and unequivocally determined that D2R forms transient dimers with a lifetime of 68 ms in its resting state. Agonist addition prolonged the dimer lifetime by a factor of ~1.5, suggesting the possibility that transient dimers might be involved in signaling.

## Introduction

G-protein-coupled receptors (GPCRs) constitute the largest protein superfamily in the human genome, including about 800 genes. They are involved in many physiological processes and are currently one of the most prominent pharmacological targets. Despite the importance of GPCRs, the fundamental question of whether GPCRs form and work as monomers, dimers, and/or greater multimers have remained unanswered [1–16]. The functional units of class-C GPCRs, including metabotropic glutamate receptor 1 [17–21] and GABA_B_ receptors [22–24], are generally considered to be constitutive homo- and/or hetero-dimers. However, our knowledge about the oligomeric states of the largest class of GPCRs, the class-A group GPCRs, is quite limited. They might exist as monomers [3, 4, 16, 25], dimers [26–32], constitutive multimers [33] and/or dynamically interchanging monomers and dimers [34–39], even in the ER membrane [37].

The first step toward resolving this issue is to determine whether class-A GPCRs form dimers and multimers in the plasma membranes (PMs) of *living cells*, rather than relying on *in vitro* experiments [34–39], and to employ methods that can directly observe the dynamic interconversions between monomers and di-/multimers. Previously, by developing a single fluorescent-molecule imaging method, the dynamic monomer–dimer equilibrium for a GPCR, the N-formyl peptide receptor (FPR), was determined in living cells, for the first time ever for any membrane molecule [35]. In quiescent cells before agonist stimulation of FPR, the dimer–monomer 2D-equilibrium constant was 3.6 copies/μm^2^, with 2D-dissociation and 2D-association rate constants of 11.0 s^−1^ and 3.1 [copies/μm^2^]^−1^s^−1^, respectively, at 37 °C. With the physiological FPR expression level of ~2.1 receptor copies/μm^2^ (~6000 copies/neutrophil), monomers continually convert into dimers every 150 ms, dimers dissociate into monomers in 91 ms (dimer lifetime; inverse the 2D-dissociation rate constant), and at any moment, 2500 and 3500 receptor molecules participate in transient dimers and monomers, respectively. Transient homodimers of the class-A GPCR were also found for M1 muscarinic receptor [34], β1-adrenergic receptor (AR), β2-AR [36], and dopamine D2 receptor (D2R) [39]. Interestingly, in the case of FPR, even after stimulation of the antagonist, the monomer–dimer equilibrium was not affected [35].

In the present study, to advance this line of research further, we further examined whether a class-A GPCR, D2R, forms transient or prolonged dimers and whether ligation influences dimerization. We selected D2R because it plays a critical role in the development of psychosis, as illustrated by the fact that many antipsychotics act by modulating D2R. Furthermore, D2R dimerization might be involved in the pathophysiology of schizophrenia as well as in the sensitized state after exposure to amphetamine, a drug that can cause psychosis [40]. Furthermore, D2R might play important roles in other tissues: in addition to neurons in the cerebral cortex, olfactory tubercle, and hippocampus, D2R is ubiquitously expressed in various tissues, including blood vessels, adrenal gland, heart, and kidney [41].

D2R homo-dimerization has been a controversial subject matter. On the one hand, constitutive dimerization was reported [42], but on the other hand, transient dimerization was suggested [11, 39, 43]. Meanwhile, agonist stimulation was found to reorient the fourth transmembrane helix in the constitutive dimer [42], suggesting that allosteric communication between the protomers of D2R dimers modulated the protomer activation [44]. Furthermore, the presence of monomers and various multimers up to pentamers of D2R has been reported [39, 45].

Previously, using single-molecule imaging, Tabor et al. [39] detected transient D2R homodimers with a lifetime of ~0.5 s, which was longer than that we found for FPR (91 ms) by a factor of 5.5. It should be noted that our experiments for FPR were performed at 37 °C, whereas the result for D2R dimer lifetime by Tabor et al. was obtained at 24 °C. Meanwhile, other data of GPCR homodimer lifetimes were all conducted at lower temperatures: M1 muscarinic receptor (lifetime ~0.7 s, 8x longer than FPR dimer lifetime) at 23 °C [34] and β1- and β2-AR (lifetime ~5 s, 55x longer than FPR dimer lifetime) at 20.5 °C. Due to such variations, we consider that the GPCR homodimer lifetimes should be better defined, by observing them at a physiological temperature of 37 °C.

Furthermore, to investigate the physiological relevance of GPCR homodimers, the effect of agonist on the homodimer lifetime would be quite important. The results about this point reported in the literature are quite varied. No effects were detected on the homodimers of FPR [6, 35], β1- and β2-AR [30, 36, 46], M1 muscarinic receptor [30], 5HT2c [47] and 5HT4 [26] serotonin receptor, and neurotensin receptor 1 [33], as well as β1–β2 AR heterodimers [46]. On the other hand, an increase of apparent dimer fraction was observed for D2R upon agonist addition [39], which might be due to the agonist-induced rearrangements of the transmembrane α-helices within the D2R homodimers [48] (similar findings were made for CCR2 [49] and CXCR4 [49]).

Here, using single fluorescent-molecule imaging-tracking, we unequivocally detected transient D2R dimers in the live-cell PM, and determined their lifetimes at 37 °C. Furthermore, we found that the D2R dimer lifetimes were prolonged after agonist stimulation.

## Materials and Methods

### Cell Culture, cDNA Construction, and Expression in CHO-K1 Cells

CHO-K1 cells, which do not respond to dopamine stimulation [50], were cultured in Ham’s F12 medium (Sigma) supplemented with 10% (v/v) FBS, 100 units/ml penicillin, and 0.1 mg/ml streptomycin. The cells were transfected with the cDNA encoding acyl-carrier protein (ACP)-tagged human D2R (cDNA encoding the short variant; Addgene) in the plasmid vector pcDNA3.1(+) (Life Technologies) using LipofectAMINE PLUS (Life Technologies), according to the manufacturer’s recommendations. Transfected cells were seeded in glass-base dishes (35-mmϕ with a glass window of 12 mmϕ, 0.12–0.17-mm-thick glass; Iwaki), and cultured for 24–48 h before use. ACP is known to be monomeric [51].

The treatments of the cells with agonists and neutral antagonists were performed by adding 1 ml solutions of 30 µM dopamine (Wako), 30 µM (-)-quinpirole (SIGMA-Aldrich) or 200 nM (+)-UH-232 (TOCRIS), in Hank’s balanced salt solution (HBSS, Nissui) buffered with 2 mM piperazine-N,N’-bis(ethanesulfonic acid) (PIPES) from Dojindo (Kumamoto, Japan), at pH 7.4 (P-HBSS).

### Labeling of D2R with the Fluorescent Dye ATTO594

ACP-tagged D2R expressed in the PM was labeled with ATTO594 (ATTO-TECH), by incubating the cells with 2 µM ATTO594-Coenzyme A (custom-synthesized by Shinsei Kagaku, Osaka, Japan, by conjugating ATTO594-maleimide to Coenzyme A-SH [New England Biolabs]) and 1 µM phosphopantetheine transferase at ~25 °C for 20 min, which are the conditions that achieved ~95% labeling of ACP [52]. The cells were washed with P-HBSS three times, and then the washed cells were subjected to microscopic observations. The cells exhibiting the presence of 0.4–0.8 ATTO594-ACP-D2R spots/µm^2^ were used for single-molecule imaging experiments.

### Single Fluorescent-Molecule Video Imaging, Detection of Colocalization-Codiffusion, and Determination of Dimer Lifetimes

Single molecules of the fluorescently-labeled ACP-D2R expressed in the bottom PM were observed at 37 °C, using a home-built objective-lens-type total internal reflection microscope based on an Olympus IX-70 inverted microscope [53, 54], with a 100x objective lens (numerical aperture of 1.49; Olympus UAPON100XOTIRF). Fluorescent images were projected onto a two-stage microchannel plate intensifier (C8600-03, Hamamatsu Photonics), coupled to a scientific complementary metal–oxide–semiconductor camera operated at 30 or 250 Hz (C11440-22CU, Hamamatsu Photonics). The acquired image sequence was directly recorded on a solid-state drive installed on a personal computer. Fluorescent spots were identified by using a home-made computer program, as described previously [55].

Colocalization of two fluorescent spots (molecules) of the same color (the same ATTO594 probe) was detected when the two fluorescent molecules became localized within 220 nm from each other [35, 54]. An image-correlation analysis revealed a threshold value of 220 nm for detecting one or two peaks (for Alexa594 and DY547, 219 ± 9.0 nm, Kasai et al. [35]). Obviously, a scale of 220 nm is much greater than the molecular scale. Therefore, in addition to the colocalization due to the specific direct/indirect molecular binding as well as the entrapment in the same nano-domain, the incidental approaches of molecules within 220 nm, termed “incidental colocalization”, will often occur. However, although unassociated molecules may track together by chance over a short distance, the probability of this occurring for multiple frames is small, and therefore, longer colocalization durations imply the presence of molecular interactions between the two molecules, rather than incidental approaches within 220 nm (see the Results section). A similar method for detecting colocalization-codiffusion has been proposed [56].

Each time a colocalization-codiffusion event of D2R molecules was detected, its duration was measured, and after many such colocalization events were observed, a histogram showing the distribution of colocalization durations was obtained. The histogram could be fitted by a single exponential function, which provided a decay constant (lifetime; *τ*_observed_). This value was then corrected for the photobleaching lifetime (*τ*_bleaching_) to obtain the duration of apparent colocalization (*τ*_apparent colocalization_), using the equation [*τ*_observed_^*−*1^−2*τ*_bleaching_^−1^]^−1^ = *τ*_apparent colocalization_ [35, 57]. (In our previous work [35], by comparing the results obtained by recordings at a 4-ms time resolution, we showed that *τ*_observed_ of 110 ms could be determined precisely by 33-ms-resolution observations [normal video rate]. Therefore, we employed normal-video-rate observations in the present study.)

In principle, since the histogram of individual dimer durations should include the contributions of both specific and incidental colocalization events, the durations of which are described by complex functions. Nevertheless, we found that the incidental colocalization duration distribution could be quite-well described by a single exponential function with an incidental colocalization lifetime of 19 ± 0.66 ms, using a monomer reference molecule, ACP linked to the transmembrane domain of the low-density lipoprotein receptor, called ACP-TM [35]. Furthermore, as described in the previous paragraph, the distribution of colocalization durations could be fitted by a single exponential function, probably due to the insufficient time resolution and/or poor signal-to-noise ratios, suggesting that its lifetime, *τ*_apparent colocalization_, closely represents the distribution of the sum of the durations of the following three events: duration from the encounter of two molecules within the distance of 220 nm until the binding of two molecules (duration 1), that of actual binding of two molecules (duration 2), and that from the separation of two molecules until they become separated over the distance of 220 nm (duration 3). Therefore, at the time resolution and signal-to-noise ratio of the present instrument, the best estimate of the binding duration of the two molecules would be *τ*_apparent colocalization_ minus incidental colocalization lifetime of 19 ± 0.66 ms (assuming that this lifetime is close to the sum of duration 1 and duration 3). The pure incidental colocalization events are not included in this argument, because the fitting was performed from the second bin (33 ms and longer), which excludes the majority of incidental events.

Although we tracked the signal intensities as well as the (x, y)-coordinates of single molecules, we used the signal intensity information only for the purpose of confirming colocalization. In our single-molecule tracking with the signal intensity, we hardly observed any spots exhibiting two-step photobleaching. This observation excludes the possibility that we missed fluorescent spots of dimers that last for very long durations (if we had tracked colocalization using only the (x, y)-coordinates of single molecules, long lasting dimers might appear like single molecules, and thus we might miss the long-lasting dimers). Meanwhile, even when two fluorescent spots partially overlapped, although the signal intensities of such spots might appear to be those of dimers, the two molecules were often found to be separated from each other by a distance longer than 220 nm (thus, no colocalization, and thus no binding), and therefore, simple intensity measurements were not useful for unequivocally detecting colocalization.

## Results

### D2R Molecules Exhibited Transient Colocalization-Codiffusion

All single-molecule tracking experiments were performed at 37 °C, at a time resolution of 33 ms (video rate) or 4 ms. D2R conjugated at its N-terminus with ACP was expressed in CHO cells, which do not express endogenous dopamine receptors, and D2R in the PM was fluorescently labeled by covalently conjugating ATTO594. This method allowed almost complete fluorescent labeling of D2R (>95%, George et al. [52]). The expression levels of D2R in the PM were adjusted to 0.4–0.8 copies/µm^2^.

**Fig. 1:**
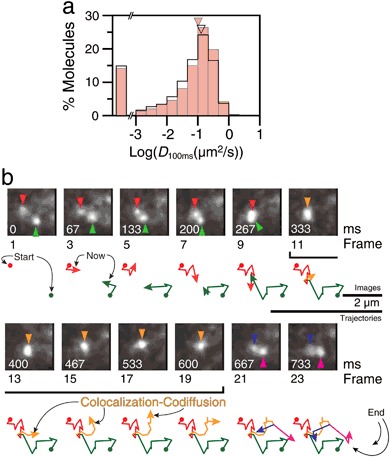
D2R molecules continually form transient dimers. **a** The histograms showing the distributions of the diffusion coefficients evaluated in the interval between 67 and 133 ms (*D*_100ms_), before (pink bars) and 20–120 s after dopamine (agonist) stimulation (black open bars). Since even the ATTO594 molecules bound to the glass exhibit apparent mobility due to noise, with apparent *D*_100ms_ up to 0.01 µm^2^/s, D2R molecules exhibiting apparent *D*_100ms_ greater (smaller) than 0.01 µm^2^/s were defined as (im)mobile molecules. The percentages of immobile molecules were practically unchanged (20%) after the addition of dopamine. The triangles indicate the median values including immobile D2R molecules: 0.10 and 0.084 µm^2^/s before and after stimulation, respectively. The median values excluding immobile molecules (molecules with *D*_100ms_ smaller than 0.01 µm^2^/s) were 0.14 and 0.13 µm^2^/s before and after stimulation, respectively. Before and after stimulation, 2183 and 2595 molecules in six cells were inspected, respectively (no statistically significant difference after dopamine addition, using the Brunner–Munzel test). **b** A typical video sequence and trajectories of single D2R molecules undergoing temporary colocalization-codiffusion, recorded at a 33-ms resolution (video rate; without ligation). The two molecules (red and green triangles in the images and trajectories) first became colocalized in video frame 11 (333 ms), diffused together for 10 video frames (333 ms, yellow trajectories; Colocalization-Codiffusion), and then separated (blue and magenta triangles and trajectories)

The D2R molecules in the PM were observed at the level of single molecules. In quiescent cells, as shown in Figs. 1a and b, most D2R molecules (~80%) were mobile, exhibiting a median diffusion coefficient of 0.10 µm^2^/s in the time windows of 67 and 133 ms (*D*_100ms_) (Fig. 1a; 0.14 µm^2^/s when only mobile D2R molecules were examined; see the caption to Fig. 1a for details). The typical images and trajectories displayed in Fig. 1b indicate that D2R molecules undergo diffusion and frequent transient colocalization-codiffusion with other D2Rs, and such colocalization-codiffusion occurred frequently throughout the PM observed here.

### Durations of Colocalization-Codiffusion of Two D2R Molecules

Since each fluorescent spot representing a single fluorescent molecule is much larger than the molecule itself (~500 nm vs. ~5 nm in diameter), even unassociated molecules become colocalized without molecular interactions and might track together by chance over a short distance. However, the probability of such incidental codiffusion events occurring for multiple frames is small, and therefore, longer colocalization implies the occurrence of molecular interactions between two molecules. Previously, we employed a monomer-reference molecule, ACP linked to the transmembrane domain of the low-density lipoprotein receptor, ACP-TM, and found that the duration of incidental colocalization-codiffusion of ACP-TM is 19 ± 0.66 ms [35].

**Fig. 2:**
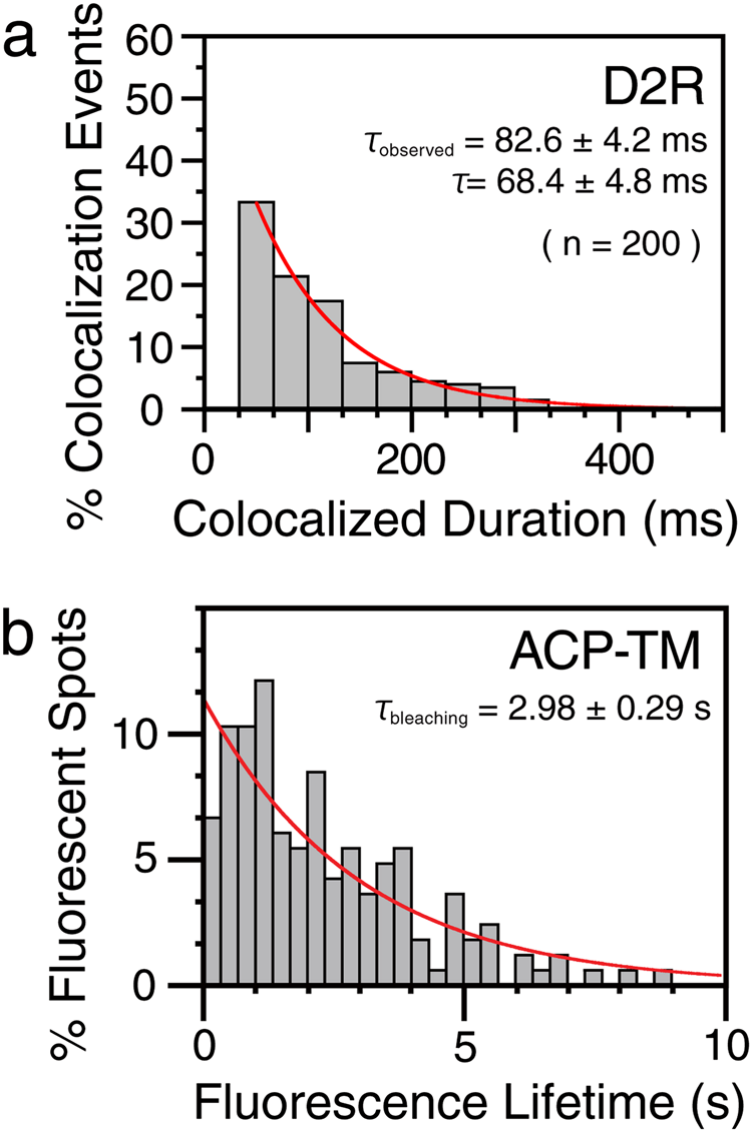
D2R dimer lifetime determined by single-molecule tracking. **a** Histograms showing the distributions of the durations of individual colocalization events for D2R and ACP-TM, observed at a time resolution of 33 ms. See Materials and Methods for details. **b** The distribution of photobleaching lifetimes of ATTO594, providing an exponential photobleaching lifetime of 2.98 ± 0.29 s (*n* = 165 molecules) by single exponential fitting. The determination was made with ACP [ATTO594]-TM expressed in the PM of CHO cells. By single-exponential fitting, the exponential lifetime for D2R colocalization (*τ*_observed_) was found to be 82.6 ± 4.2 ms (*n* = 200 colocalization events). This value was corrected for photobleaching, using the photobleaching lifetime of 2.98 s, providing a corrected colocalization lifetime (*τ*_apparent colocalization_) of 87.4 ± 4.7 ms. Since this value is the sum of the actual binding time (*τ*) and the duration for the incidental approach (+separation), which is 19 ms, the D2R homodimer lifetime *τ* was estimated to be 68.4 ± 4.8 ms (87.4–19 ms)

In the present research, each time we found a colocalization-codiffusion event of D2R molecules, we measured its duration, and after observing many such colocalization events, we obtained the distribution of colocalization durations (Fig. 2a). The histogram could be fitted by a single exponential function, providing a decay constant (lifetime; *τ*_observed_) of 82.6 ± 4.2 ms (Fig. 2a) (throughout this report, the fitting error of the 68.3% confidence limit is given as the fitting parameter error, which equals the standard error of the determined parameter).

The decay constant was corrected for the photobleaching lifetime of the fluorescent probe ATTO594 (2.98 ± 0.29 s; Fig. 2b), providing 87.4 (87 ms for two significant digits) ± 4.7 ms after correction for its photobleaching lifetime. For further discussions of the colocalization lifetimes, see Materials and Methods (Single fluorescent-molecule video imaging, detection of colocalization-codiffusion, and determination of dimer lifetimes).

### D2R Forms Homodimers, with a Lifetime of 68 ms

As the colocalization lifetime of D2R (87 ms) is much longer than that of ACP-TM (19 ms; Kasai et al. [35]) (*P* = 2.2 × 10^−16^, Brunner–Munzel test), we concluded that the D2R monomers interact with each other; i.e., D2R forms transient homodimers. Since the colocalization lifetime of ACP-TM (19 ms) represents the duration of incidental approaches of two molecules without specific molecular interactions, the actual binding duration (dimer lifetime; *τ*) of D2R molecules can be estimated by subtracting 19 ms from 87 ms, providing 68 ms (see Materials and Methods). Note that, because this duration might be the result of many dissociation-rebinding events, this number should be considered to be the collective lifetime when the dimerization occurred. Dimer lifetimes, *τ*’s, observed under various conditions are summarized in Table 1.

**Table 1:** Summary of the homodimer lifetimes of D2R before and after the addition of agonist or neutral antagonist

	Dimer lifetime (ms)	# of events	P (vs. D2R before ligation)
Before ligation	68.4 ± 4.8	200	N.A.
+Dopamine (natural agonist)	98.6 ± 8.3	232	0.0328
+Quinpirole (agonist)	103.5 ± 9.3	211	0.0484
+UH-232 (neutral antagonist)	70.5 ± 11.0	200	0.933

We previously found that the FPR homodimer lifetime was ~ 90 ms. Therefore, the lifetime of the transient homodimers of D2R is comparable to that of FPR homodimers, but slightly (25%) shorter. Previously, using single-molecule imaging at a lower frame rate (19.32 frames per second vs. 30 frames per second employed here), Tabor et al. [39] detected transient D2R homodimers with a lifetime of ~0.5 s at 24 °C, which was longer than that found here (68 ms at 37 °C) by a factor of 7.4.

We hardly detected any oligomers greater than dimers under the expression levels employed here (0.4–0.8 ATTO594-ACP-D2R spots/µm^2^ in the PM). At much higher expression levels, oligomers might form. To the best of our knowledge, the physiological expression levels of D2R have not been reported.

### Agonist Stimulation Slightly Stabilized Transient Dimers, in Contrast to a Neutral Antagonist

The effects of agonists on the D2R homodimer lifetime was investigated by incubating the cells expressing D2R in the presence of agonists (15 µM dopamine or 15 µM quinpirole) [58], or a neutral antagonist (0.1 µM UH-232) [59] for 20–120 s. This incubation time was selected because intracellular Ca^2+^ mobilization was detected within 20 s after agonist addition (15 µM; no intracellular Ca^2+^ mobilization was found after the addition of a neutral agonist, UH-232, for 2 min).

**Fig. 3:**
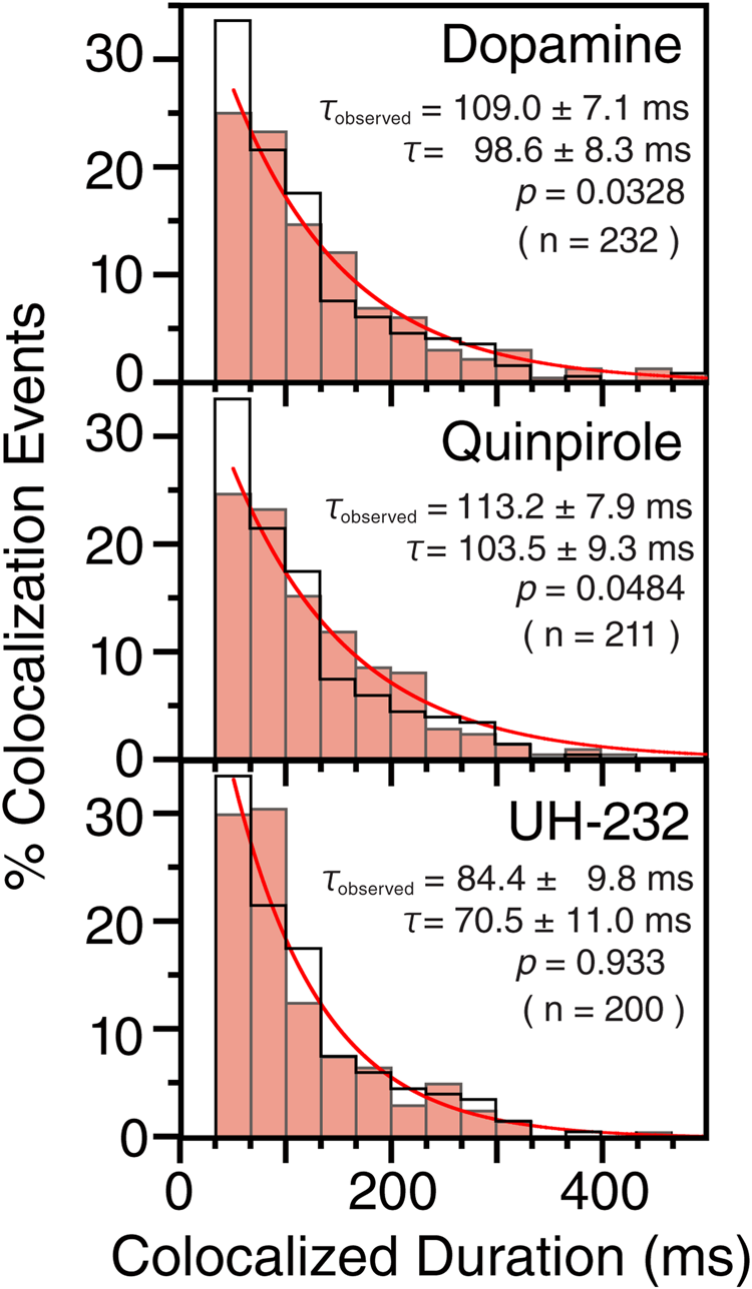
D2R dimer lifetimes after treatments with the D2R agonists and neutral antagonist. Histograms showing the distributions of the durations of individual colocalization events for D2R 20-120 s after the treatments with the D2R agonists, dopamine and quinpirole, and the neutral antagonist, UH-232 (pink bars), as compared with those before ligation (black open bars; same as those shown in Fig. 2a). The values of *τ*_observed_ were determined to be 109.0 ± 7.1 ms, 113.2 ± 7.9 ms, and 84.4 ± 9.8 ms, after the additions of dopamine, quinpirole, and UH-232, respectively. After corrections for the photobleaching lifetime and subtraction of the incidental colocalization lifetime (19 ms), the *τ* values for D2R homodimers were determined to be 98.6 ± 8.3 ms, 103.5 ± 9.3 ms, and 70.5 ± 11.0 ms after the additions of dopamine, quinpirole, and UH-232, respectively

The duration histograms for D2R colocalization-codiffusion before and after the incubations of the cells with these compounds are shown in Fig. 3. After single exponential fitting of these histograms, correction for probe photobleaching, and then subtraction of the incidental overlapping lifetime of 19 ms, the D2R homodimer lifetimes (*τ*) were determined to be 98.6 ± 8.3 ms, 103.5 ± 9.3 ms, and 70.5 ± 11.0 ms after the additions of dopamine, quinpirole, and UH-232, respectively. Namely, agonist stimulation significantly prolonged the lifetimes by a factor of ~1.5, whereas the neutral antagonist did not affect the D2R homodimer lifetime (*P* = 0.033, 0.048, and 0.93, respectively, using the Brunner–Munzel test; summarized in Table 1).

## Discussion

Whether a class-A GPCR D2R forms dimers, and if so, whether the dimers are transient or constitutive dimers, have been controversial subjects [42, 43]. In the present research, our single-molecule imaging-tracking at a time resolution of 33 ms unequivocally showed that D2R forms transient homodimers with a lifetime of 68 ms at 37 °C. This result is quite consistent with a homodimer lifetime of 90 ms for FPR observed by us at 37 °C [35]. Meanwhile, the D2R homodimer lifetime was found to be much longer (~0.5 s) at 24 °C [39], a factor of 7.4 longer than that found here. Other class-A GPCRs, M1 muscarinic receptor and β1- and β2-AR, were also found to form transient homodimers with longer lifetimes of 0.7 s at 23 °C [34] and 5 s at 20.5 °C [36], respectively. Taken together, transient homodimerization is likely to be a general physical property of the class-A GPCRs, and thus homodimers may play some important roles in GPCR signaling and/or regulation. In addition, because temperature strongly affects the homodimer lifetime of D2R (between a lower temperature of 24 °C and the physiological temperature of 37 °C) and probably those of other GPCRs, the activation energy for homodimer formation is quite large (Q10 for D2R is likely to be much greater than 3, and that for β1- and β2-AR might reach 30 or more).

Furthermore, we found that the two agonists for D2R both prolonged the homodimer lifetime, whereas the neutral antagonist did not. The increase in the homodimer lifetime by the agonist binding was only by a factor of ~1.5, but if each subunit of the dimer participates in the downstream signaling, then the signal emitted by the D2R homodimer would increase by a factor of 2.3 (1.5 × 1.5). These results are quite consistent with the finding by Tabor et al. [39], in which agonist addition increased apparent dimer fraction of D2R. In previous analyses, the addition of agonists for FPR [35] and β1- and β2-AR [36] did not change the homodimer lifetimes, and therefore, this is the first example in which the dimer lifetime was increased by agonist addition. Although the biological significance of these differences among GPCRs is not clear, our results are consistent with the findings reported by Wang et al. [40], in which schizophrenia, an amphetamine-induced sensitized state, and acute amphetamine exposure were found to enhance dimerization of D2R without altering its expression levels.

Meanwhile, a pull-down assay after chemical crosslinking revealed that another class-A GPCR, CCR2, was monomeric before stimulation, but agonist addition induced dimer formation [60]. Further studies on the effects of agonists on GPCR homodimerization are required, based on the present understanding that class-A GPCRs are likely to form transient homodimers with lifetimes shorter than 100 ms.
